# A New Species of *Comptonia* (Myricaceae) from the Early Miocene of Central Inner Mongolia, China, and Phytogeographic History of Sweet–Fern

**DOI:** 10.3390/biology11091326

**Published:** 2022-09-07

**Authors:** Deshuang Ji, Liang Xiao, Liyan Guo, Xiangchuan Li, Zeling Wu, Jiaqi Liang, Meiting Wang, Xiaoyuan Xia, Nan Sun, Chaofeng Fu

**Affiliations:** 1 School of Earth Science and Resources, and Key Laboratory of Western Mineral Resources and Geological Engineering of the Ministry of Education, Chang’an University, Xi’an 710054, China; 2 State Key Laboratory of Palaeobiology and Stratigraphy, Nanjing Institute of Geology and Palaeontology, Chinese Academy of Sciences, Nanjing 210008, China; 3 Shaanxi Key Laboratory of Early Life and Environments, Northwest University, Xi’an 710069, China

**Keywords:** *Comptonia*, the early Miocene, Inner Mongolia, China, the Hannuoba Formation, phytogeography

## Abstract

**Simple Summary:**

The deciduous shrub *Comptonia* is a monotypic genus of Myricaceae, which currently is distributed only in eastern North America, with a smaller range than that in other periods of the Cenozoic. By analyzing the macroscopic and microscopic characteristics of the leaves, we describe a new species of *Comptonia* (i.e., *Comptonia hirsuta*) from the Hannuoba Formation in Zhuozi, Inner Mongolia, North China. The co-occurring fruits were also studied based on their morphological characteristics, which were assigned to *Comptonia tymensis*. Variation in the distribution range of *Comptonia* indicates the influence of global cooling on the expansion of this plant. Furthermore, the Bering Land Bridge played an important role in the migration from North America to East Asia. The Thulean route may have provided an opportunity for plant exchange between western Europe and eastern North America. Moreover, the reason for the disappearance of *Comptonia* from China according to the analysis of the changes in both the global climate and the distribution of *Comptonia* fossils is also discussed. It is suggested that the climatic changes after the late Miocene and the progenitive pattern of *Comptonia* together caused the disappearance of *Comptonia* in China.

**Abstract:**

*Comptonia* (Myricaceae) is well known as a monotypic genus living only in eastern North America; however, fossils show that the genus occurred extensively in the Northern Hemisphere during the Cenozoic. We observed dozens of *Comptonia* leaf fossils from the early Miocene in Zhuozi, China. The leaf architecture characteristics and epidermal features of the fossil specimens are described in detail here for the first time, and they were assigned to a new species: *Comptonia hirsuta*. The fruit fossils collected simultaneously from the same layer were assigned to *Comptonia tymensis*. The global fossil records indicate that the spatial distribution range of *Comptonia* reached its peak in both the Eocene and Miocene as two warm periods and then gradually decreased in the Oligocene, as well as after the late Miocene, because of the cooling global climate. Furthermore, the *Comptonia* taxon in East Asia may have migrated from North America via the Bering route in the late Paleocene or Eocene. Plant exchange between western Europe and eastern North America possibly occurred during the Eocene via the Thulean route. Phytogeographic variation in the *Comptonia* fossils from China also indicates that the reason for the disappearance of *Comptonia* from China may not only be due to the prolonged cooling and drying after the late Miocene, but also due to its progenitive pattern.

## 1. Introduction

*Comptonia* L’Héritier de Brutelle ex Aiton is a monotypic genus of Myricaceae, which belongs to a kind of deciduous low shrub. It lives in the habitats with dry, sterile, sandy to rocky soils in pinelands or pine barren lands, clearings, or the edges of woodlots [1]. At present, only three taxa of *Comptonia* survive, including a single species, *Comptonia peregrina* (Linnaeus) Coulter, and two varieties, *C. peregrina* var. *tomentosa* A. Chevalier and *C. peregrina* var. *aspleniifolia* (L.) Fernald. They are presently limited to North America with a distribution range extending from Nova Scotia to North Carolina, western South Carolina, northern Georgia, and west to Saskatchewan, Minnesota, Illinois, and Tennessee [2,3,4].

Although the extant *Comptonia* populations live only in North America, the fossil records of this species indicate that it was once widely distributed in the Northern Hemisphere during the Paleogene and Neogene [3,5,6,7,8,9,10,11,12,13,14,15,16,17,18,19,20,21,22,23,24,25,26,27,28,29,30,31,32,33,34,35,36,37,38,39,40,41,42,43]. Because of its distinctive leaf type, the earliest discovered fossils of *Comptonia* were incorrectly assigned to *Aspleniopteris* by Sternberg in 1825 [3,44]. Afterwards, Brongniart pointed out Sternberg’s error and then classified the fossils previously misidentified by Sternberg into *Comptonia* L’Héritier de Brutelle ex Aiton in 1828 [45]. Since then, *Comptonia* fossils have begun to attract more attention from researchers. Subsequently, an increasing number of fossils of this genus have been discovered and studied [3,5,6,7,8,9,10,11,12,13,14,15,16,17,18,19,20,21,22,23,24,25,26,27,28,29,30,31,32,33,34,35,36,37,38,39,40,41,42,43]. Previous researches indicated that *Comptonia* appears to have originated in the Arctic during the Late Cretaceous and became widely distributed in the Northern Hemisphere during the Paleogene and Neogene with its dispersals [3], such as the late Eocene, Oligocene, Miocene, and late Pliocene in Russia [40]; the Paleogene and Neogene in North America [30,41]; the Miocene in Denmark [10,42]; the Oligocene to Miocene in Germany [43]; the Oligocene to Miocene in Bohemia and other parts of Europe [38]; and the late Eocene to Miocene in China [6,23,26,35,36,37].

In this paper, we describe a new species of *Comptonia* based on 93 recently collected leaves from the early Miocene Hannuoba Formation in Zhuozi County, Ulanqab City, Inner Mongolia Autonomous Region, North China. The new species is compared in detail with living equivalents and previously reported fossils on the gross morphology and cuticular microstructure of the leaves. The fruit fossils collected simultaneously from the same layer are identified according to the nutlet features. In addition, according to the global fossil records of *Comptonia* and global climate change during the Cenozoic, the reason for changes in the distribution of the *Comptonia* population during the Cenozoic is inferred. Finally, the reason for the disappearance of the *Comptonia* population from China is discussed.

## 2. Materials and Methods

### 2.1. Materials

*Comptonia* fossil leaves (ca. 93 pieces) and fruits (ca. 6 pieces) were collected from the Hannuoba Formation in Zhuozi County (40°45′01′′ N, 112°48′46′′ E), Ulanqab City, Inner Mongolia Autonomous Region, North China (Figure 1). This formation is mainly composed of olivine, pyroxene, and bubble basalts with fluvial and lacustrine sedimentary interlayers, which are known as Hannuoba basalts [46]. At this site, the fossiliferous layers include gray mudstones and black oil shales, yielding rich and well-preserved animal and plant fossils. In addition, the Hannuoba Formation was dated between 19.35 Ma and 22.84 Ma based on basalt K-Ar dating from Zuoyun County in Shanxi Province adjacent to Inner Mongolia [47]. Thus, the fossiliferous layer herein is considered to date as the early Miocene.

To compare current fossils with living *Comptonia peregrina*, the extant leaf specimens were collected from Virginia, Maine, Massachusetts, and New Hampshire in the USA. All the materials are now preserved in the Geological Museum of Chang’an University.

### 2.2. Experimental Methods

To obtain clean cuticles of the fossil leaves, we used a traditional method that was modified by Liang et al. [48]. The specimens were soaked in 20% HCl for 2 h to remove carbonate sediments and washed repeatedly with distilled water until the solution was neutral. Then, we soaked the leaves in 20% hydrofluoric acid for 12–24 h until the silicate deposits dissolved and washed them again until neutral. We moved the fossil leaves into 1% NaClO for 5–10 min and washed the solution to neutrality when the sample turned light yellow. Finally, the obtained leaf fossil cuticles were stained with 1% safranin stain and then mounted. Observations and photographs were performed using a Leica DM1000 light microscope (LM). Additionally, some specimens were selected and sprayed with gold for observation, and we took pictures with an FEI Quanta 650 FEG scan electron microscope (SEM).

The extant leaves were prepared with a 1:1 (v:v) solution of 60% glacial acetic acid and 30% H_2_O_2_, and then they were placed in a hot water bath at 60 °C for 8–12 h. Once the cuticles turned white and transparent, they could be easily separated. After completing the above steps, we stained the cuticles with safranine solution for 5–10 s and mounted them. For the LM and SEM preparations, the same procedures as those for the fossil leaves were then followed.

The fruit fossils were soaked in distilled water for 2 h. Then, we brushed off the mud and soaked them in 10% HCl for 12 h. We then washed them with distilled water until the solution was neutral. The specimens were soaked in 15% hydrofluoric acid for 12 h, and the solution was washed until neutral. Finally, we made observations and took photographs using a Leica M165 FC stereomicroscope.

## 3. Results

Order: Myricales.

Family: Myricaceae Richard ex Kunth (1817).

Genus: ***Comptonia*** L’Héritier ex Aiton (1789).

Species: *Comptonia hirsuta* Liang Xiao et De-Shuang Ji sp. nov.

Holotype: ELS-16-301AB (Figure 2A,C,G).

Paratypes: ELS-16-216 (Figure 2D,H), ELS-16-464 (Figure 2B), ELS-16-435 (Figure 2E), ELS-16-400 (Figure 2F,L).

Etymology: The specific epithet is based on the evident epidermal features of the fossil leaves, meaning abundant trichomes.

Diagnosis: Laminas oblanceolate to mostly narrow elliptical; lobes pinnatisect; dense simple trichomes with unicellular base or two, four, six, eight unicellular fused base; and peltate glandular trichomes on both the adaxial cuticle and the abaxial cuticle.

Type locality: Yangjuanwan Village, Zhuozi County, Inner Mongolia, North China.

Stratum and age: the Hannuoba Formation, early Miocene.

Repository: Geological Museum of Chang’an University, Xi’an, China.

Description: The leaves are simple, 20–85 mm long, and 6–22 mm wide. Their petioles are short, 0.3–0.6 cm in length, expanding towards the base. The laminas are oblanceolate to mostly narrow, elliptical, pinnatisect, with alternate to opposite lobes (Figure 2). The lobes are entire-margined, broadly attached to the midrib, and separated by sinuses reaching up to the midrib. Individual lobes are triangular, falcate, semi-oval, and taper into the apex and base. The apex of the lobes is straight or acuminate. The upper side of the lobes is straight to slightly concave, and the bottom side is straight to convex. The basal pair of lobes is decurrent on the petiole. The apical lobe is elliptical without dissection. The venation is pinnate. The midrib is simple, 0.2–0.5 mm wide in the middle of the leaf length, and tapers towards the apex. Two to four secondary veins per lobe stretch from the midrib at an angle of 80°–90°. Two of them are thicker, craspedodromous, and converging to the apex of the lobe. The other two secondary veins are eucamptodromous. There are one to three intersecondary veins parallel to the secondaries. Their lengths are similar to or more than 50% of the subjacent secondary. The intersecondary distal course is basifixed, intersecting with adjacent secondary veins. The tertiary veins consist of two types, including intercostal and epimedial tertiaries. The intercostal tertiary veins cross between adjacent secondaries, which are divided into two forms. Some of them are mixed, including opposite and alternate percurrent courses. The others are transversely freely ramified. The epimedial tertiaries are ramified, and parallel to the intercostal tertiary veins. Some of the leaves show unobvious quaternary veins that are irregularly reticulate.

The epidermal anatomy of the above described leaves is well preserved. The adaxial cuticle is hairy, showing outlines of cells ca. 19.8–39 μm in length and ca. 15.6–21 μm in width, demarcated by almost straight to wavy anticlinal cell walls (Figure 3). The abaxial cuticle is hairy, showing outlines of cells ca. 14.6–21.4 μm in length and ca. 9.9–12.1 μm in width, and anomocytic, broadly elliptical stomata are 11–16 × 13–24 μm in size (Figure 4). Unicellular simple trichomes appear on both sides of the leaves, and their unicellular bases are surrounded by six to eight cells (sometimes two, four, six, or eight of the unicellular bases are fused; see Figure 3, Figure 4, Figure 5 and Figure 6). There are abundant trichomes on the abaxial cuticle. The glandular trichomes are peltate with the originally globular balloon-shaped heads on both the adaxial cuticle and the abaxial cuticle. The head cells of the peltate glandular trichomes are disc-shaped and compressed due to the process of fossilization, 50–110 μm in diameter (Figure 3A,E,G; Figure 4E,G; Figure 5I; Figure 6D,E,H,I). The simple stalks of the peltate glandular trichomes are 12–15 μm in diameter.

Comparison: The above described leaf materials from the Zhuozi County, Inner Mongolia, North China, can be easily assigned to the genus *Comptonia* based on the typical pinnately lobed leaves with entire-margined lobes. The foliage of *Comptonia hirsuta,* as described above, has some common features with records from the Paleogene and Neogene in Czechia [38] and Weichang [36], including both gross morphology and epidermal features, such as the shape of the laminas, the features of veins, and the stomatal apparatus type. However, *C. hirsuta* has denser trichomes, more diverse forms of the lobes, and larger head cells of peltate glandular trichomes than those of *C. naumannii*. Moreover, the size of the foliage of *C. difformis* is larger than that of *C. hirsuta* in general, while the size of the head cells of the peltate glandular trichomes are smaller. In addition, the simple trichomes and peltate glandular trichomes are on both the adaxial cuticle and abaxial cuticle of *C. hirsuta*, while those of *C. difformis* were not observed on the adaxial cuticle because of the poorly preserved epidermal structure from Czechia [38]. The differences between *C. hirsuta* and late Eocene *C. anderssonii* Florin (1920) from Fushun, China, include the type of the stalks of the peltate glandular trichomes and the size of the laminas. *C. hirsuta* has only the unicellular stalks of the peltate glandular trichomes, while two-celled, three-celled, and four-celled stalks of the peltate glandular trichomes appear in *C. anderssonii*. Additionally, the size of the laminas in *C. hirsuta* is visibly larger than that of the laminas of *C. anderssonii*. Furthermore, *C. hirsuta* can be easily distinguished from *C. columbiana* [30,37,41] by the serrate leaf margin of the latter. Meanwhile, *C. hirsuta* has larger head cells of the peltate glandular trichomes than those of Eocene *C. columbiana* from Fushun and Yilan, China. Sadowski et al. [39] reported some amber fossil leaves assigned to *Comptonia* from Samland Peninsula near Kaliningrad (Russia) in the late Eocene (34–38 Ma) with head cells of the peltate glandular trichomes as large as the fossil leaves in this research. However, the former species has circular to elliptical trichome bases composed of up to 13 radially arranged cells, which are different from those of *C. hirsuta*. Although many fossil leaves have been described as *Comptonia* throughout geological history, none of the other fossil species confirmed the anticipated affinity to this genus according to the epidermal anatomical characteristics. Therefore, we prefer to identify the *Comptonia* fossil collected in Zhuozi County as a new species, namely, *C. hirsuta*, on account of the dense simple trichomes with a unicellular base or two, four, six, or eight unicellular fused base and the peltate glandular trichomes on both the adaxial cuticle and abaxial cuticle.

Species: *Comptonia tymensis* Dorofeev.

1994 *Comptonia tymensis* Dorofeev, Budantsev, p. 33, pl. 70, figs. 1–19.

2010 *Comptonia tymensis* Dorofeev, Liang et al., p. 55, pl. 5.

Material: ELS-18-116, ELS-18-205, ELS-18-154, ELS-18-246, ELS-18-232, ELS-16-432.

Description: Nutlets are elliptic to ovoid, 3.1–4.4 mm long and 1.6–2.9 mm wide. The length/width ratio is 1.42–1.87, the apex is acute or acuminate, and the base is widely cuneate to rounded with a knob-like blunt scar. The external surface has four to eight conspicuous longitudinal ribs extending from the basal scar and, rarely, to the apical part of the nutlets (Figure 7). The external surface of the endocarp is smooth with four to eight ribs corresponding to those of the nutlets (Figure 7B–D).

Comparison: The fruits of this shrub can easily be classified as belonging to the genus *Comptonia* based on their typical shape. The morphological characteristics of the nutlets in *Comptonia* for identification at the species level include the size, shape, surface characteristics, and the number and characteristics of the ribs [38]. The above described fruits are similar to those of *C. tymensis* Dorofeev from the early Miocene of Weichang, except for their slightly smaller size and slightly larger number of ribs. The endocarps/nutlets of *C. srodoniowae* Friis are different from those of this research because of their shape and the style of the base. *C. srodoniowae* has a guttiform to fusiform shape and a long mucronate style base [10]. However, the fruits in this research have an elliptic to ovoid shape and a knob-like blunt scar at the base. In addition, the fruits of *C. tymensis* are also different from those of the extant *C. peregrina* because of their knob-like blunt scar.

Although the leaf specimens and co-occurring fruits were collected from the same layer of the same site in Zhuozi and seem to belong to the same species, they were not directly linked together. For the sake of caution, we did not assign the six fossil fruits and all the fossil leaves that co-occurred at the same time to the same species.

## 4. Discussion

### 4.1. Comparison between Comptonia hirsuta and C. peregrina

The fossils discovered from the Hannuoba Formation in Zhuozi, China, are similar to the living *C. peregrina* in leaf structure. They share many morphological features, such as the same pinnately lobed leaves, their leaf structure, and the similar shape of their leaves. On the other hand, they also have some common epidermal characteristics, including the anomocytic stomata on the abaxial cuticle, the dense unicellular simple trichomes, and peltate glandular trichomes on two sides of the epidermis. Teodoridis et al. [38] considered the simple and paired trichomes in *C. peregrina* to be more robust. However, the four-celled fused base also appeared in the adaxial cuticle of *C. peregrina* in our observation, and this feature can also be observed in *C. hirsuta* (Figure 3). Furthermore, there are abundant trichomes in the leaves of both extant *C. peregrina* and *C. hirsuta*. Nevertheless, there are some differences between these two species. Firstly, the size of the head cells of the glandular trichome is distinct. The size of the mature head cells of the glandular trichomes in extant *C. peregrina* is usually ca. 60 μm in diameter [49], while the head cells of the glandular trichomes on the cuticles of *C. hirsuta* can reach 50–110 μm in diameter. Additionally, the types of the stalks of the peltate glandular trichomes in extant *C. peregrina* are one-celled, two-celled, and four-celled [36], while the type of the stalks of the peltate glandular trichomes in *C. hirsuta* is only one-celled (Figure 4G, Figure 5I, Figure 6D,E,H,I).

In addition to the differences presented above, the density of trichomes in *C. naumannii* is lower than that in the two above mentioned species. Normally, plants living in arid environments have more trichomes than those living in humid environments. This may indicate that the early Miocene *C. hirsuta* of Zhuozi lived in a drier environment than the *C. naumannii* in Weichang at the same time. It is noteworthy that the similarity of the leaves of the *Comptonia* fossils from the Neogene and Quaternary (e.g., *C. naumannii*, *C. hirsuta*, and *C. difformis*) and those of the extant *C. peregrina* is very high, which may indicate that the morphological evolution of the genus, at least since the early Miocene, was relatively slow. *Comptonia* was widely distributed in the Northern Hemisphere during the Paleogene and Neogene periods. However, after the Quaternary ice age, it became a monotypic genus and its distribution was limited to North America, indicating that *Comptonia* is a relict and can also be regarded as a “living fossil”.

### 4.2. Phytogeographic History of Comptonia

*Comptonia* is a deciduous tree that does not tolerate shade and naturally grows in well-drained, dry, acidic, sandy, or gravelly clearings in coniferous forests. It is presently living in eastern North America as one of the refugia. However, the *Comptonia* population was once widely distributed in the Northern Hemisphere during the Paleogene and Neogene [3,5,6,7,8,9,10,11,12,13,14,15,16,17,18,19,20,21,22,23,24,25,26,27,28,29,30,31,32,33,34,35,36,37,38,39,40,41,42,43].

Fossil records of *Comptonia* have been found in North America (e.g., New Jersey), Greenland, and Europe (e.g., Transylvania, Central Europe, and Russia), from the Late Cretaceous [3,40]. It seems to have originated in the Northern Hemisphere at high latitudes in at least the Late Cretaceous. However, these fossils are still questionable regarding their age and their systematic identity; thus, they need to be researched in more detail [36]. In the Paleocene, reliably identified *Comptonia* only appeared in high latitudes, such as Axel Herberg Island and Alaska [21,29], which also indicates that the *Comptonia* taxon lived mainly at high latitudes in the Northern Hemisphere. It was widely distributed in the Northern Hemisphere during the Eocene, including western North America, Europe, and East Asia [5,6,8,18,23,26,27,30,33,39]. As is known, the Turgai Strait, which was the barrier between Europe and East Asia from the mid-Mesozoic to the late Eocene, limited migration between Europe and Asia [50]. Therefore, the *Comptonia* taxon in East Asia is more likely to have migrated from Alaska, North America, via the Bering route in the late Paleocene or Eocene (Figure 8) [50]. On the other hand, the fossil records in western Europe and eastern North America during the Eocene may be due to numerous biological exchanges in the Eocene by the Thulean route, as suggested by Brikiatis (2014) [51]. In the Oligocene, *Comptonia* was mainly distributed in western North America, Europe, and Asia (Figure 8). Compared to that in the Eocene, the distribution range in the Oligocene was narrowed. Moreover, there are no fossil records from high-latitude regions during the Oligocene. The change in the distribution from the Eocene to the Oligocene is possibly due to the climatic cooling that happened in the earliest Oligocene (i.e., Oi-1 Glaciation) [52]. Moreover, the distribution of *Comptonia* spread into Kazakhstan in central Asia, belonging to the Turgai region, which may be related to the closure of the Turgai Strait in the early Oligocene. In the Miocene, *Comptonia* taxa achieved another peak in their spatial distribution, which came to include North America, western Europe, East Asia, and Iceland [7,10,16,18,22,23,24,36,38,42], corresponding exactly to the warm Miocene, especially the mid-Miocene climatic optimum (i.e., MMCO) [52]. After the Miocene, the distribution range of this genus was reduced obviously, and it disappeared from East Asia. In the Pliocene, the *Comptonia* taxon only occurred in Alaska, Yukon, and Russia [19,40]. The decrease in the distribution range of *Comptonia* may be the result of the cooling global climate after the late Miocene [52]. Because of this, the *Comptonia* taxon is only currently found in eastern North America.

In summary, *Comptonia* may have originated from the Northern Hemisphere at high latitudes before the early Paleocene, and then migrated to East Asia in the late Paleocene or Eocene via the Bering route. Plant exchange between western Europe and eastern North America possibly occurred during the Eocene via the Thulean route (Figure 8). Afterwards, this genus reached its peak spatial distribution range in the warm periods of both the Eocene and Miocene and gradually decreased in the Oligocene as well as after the late Miocene. The distribution of *Comptonia* was eventually limited to eastern North America, where it occurs at present.

### 4.3. Phytogeographic Evolution of Comptonia in China

**Figure 9:**
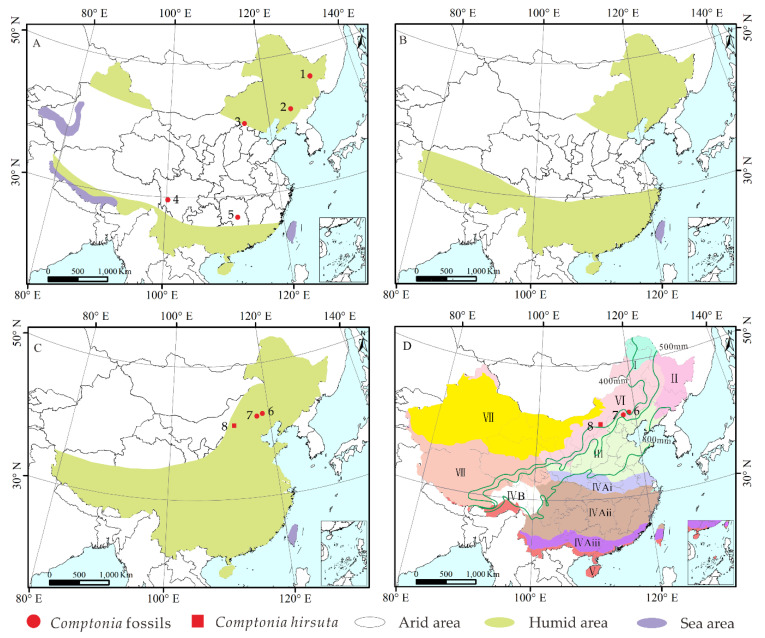
Terrestrial climate changes from the Eocene to Miocene with the megafossil records of *Comptonia* in China. (**A**) Eocene, (**B**) Oligocene, and (**C**) Miocene (redrawn with permission from Ref. [57]. Copyright 2005 Palaeogeography, Palaeoclimatology, Palaeoecology and Ref. [58]. Copyright 2021 Taylor & Fancis). (**D**) Current vegetation distribution with the respect to annual precipitation in China (redrawn from EBVMC [59]). Base maps were constructed with ArcGIS 10.2 based on data from the Resource and Environment Data Cloud Platform [53]. The Arabic numbers in (**A**,**C**) represent the sites where *Comptonia* megafossils were collected in China, respectively: 1, Eocene of Yilan, Heilongjiang; 2, Eocene of Fushun, Liaoning; 3, Eocene of Zhangjiakou, Hebei; 4, Litang, Sichuan; 5, Xiangxiang, Hunan; 6, Chifeng, Inner Mongolia; 7, Weichang, Hebei; 8, early Miocene of Zhuozi, Inner Mongolia. The green lines represent the boundary of the precipitation. The Roman numerals in (**D**) designate different climate and vegetation types: I, cold-temperate deciduous coniferous forest; II, temperate mixed coniferous and deciduous broadleaved forest; III, warm-temperate deciduous broadleaved forest; IVAi, northern subtropical evergreen and deciduous broadleaved forest; IVAii, middle subtropical evergreen broadleaved forest; IVAiii, southern subtropical evergreen broadleaved forest; IVB, subtropical alpine coniferous forest; V, tropical monsoon rainforest and rainforest; VI, temperate steppe; VII, temperate desert; VIII, Tibetan Plateau alpine vegetation.

**Figure 1:**
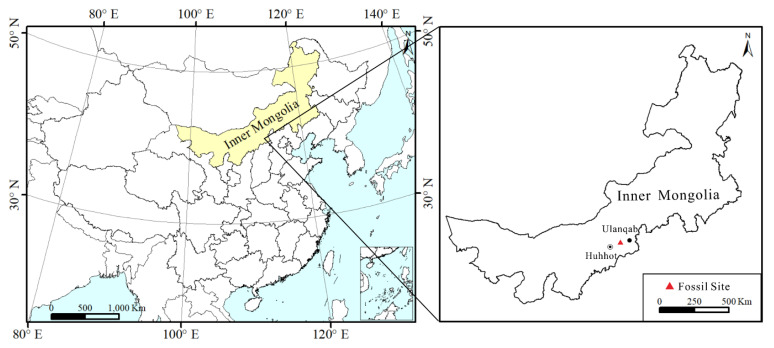
Location of *Comptonia* fossils in Zhuozi County, Ulanqab City, Inner Mongolia Autonomous Region in North China.

**Figure 2:**
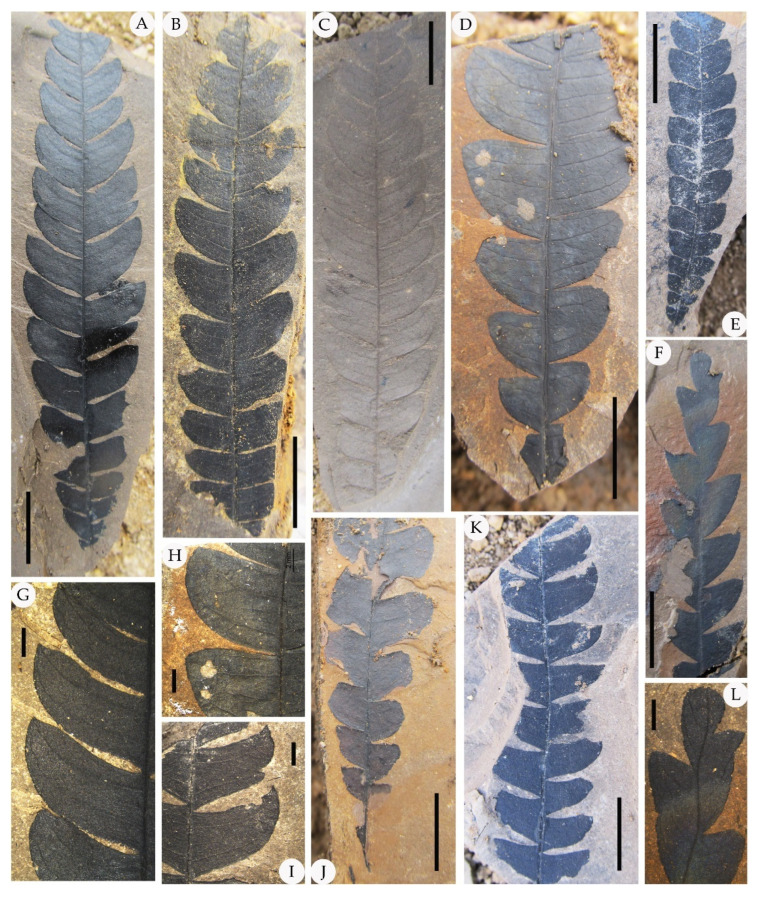
Leaf compressions and impressions of *Comptonia hirsuta* from the early Miocene of Zhuozi, North China. (**A**) Holotype, No. ELS-16-301A. (**B**) No. ELS-16-464. (**C**) Holotype, No. ELS-16-301B. The counterpart of (**A**). (**D**) No. ELS-16-216. (**E**) No. ELS-16-435. (**F**) No. ELS-16-400. (**G**) Holotype, No. ELS-16-301A. Details of (**A**) show the semi-oval lobes in the middle of the leaf. (**H**) No. ELS-16-216. Details of (**D**) show the semi-oval lobes in the middle of the leaf. (**I**) No. ELS-16-410. Details of (**K**) showing the falcate lobes in the middle of the leaf. (**J**) No. ELS-16-215. (**K**) No. ELS-16-410. (**L**) No. ELS-16-400. The top of (**F**) shows triangular lobes. Scale bar: (**A**–**F**,**J**,**K**) = 1 cm; (**G**–**I**,**L**) = 2 mm.

**Figure 3:**
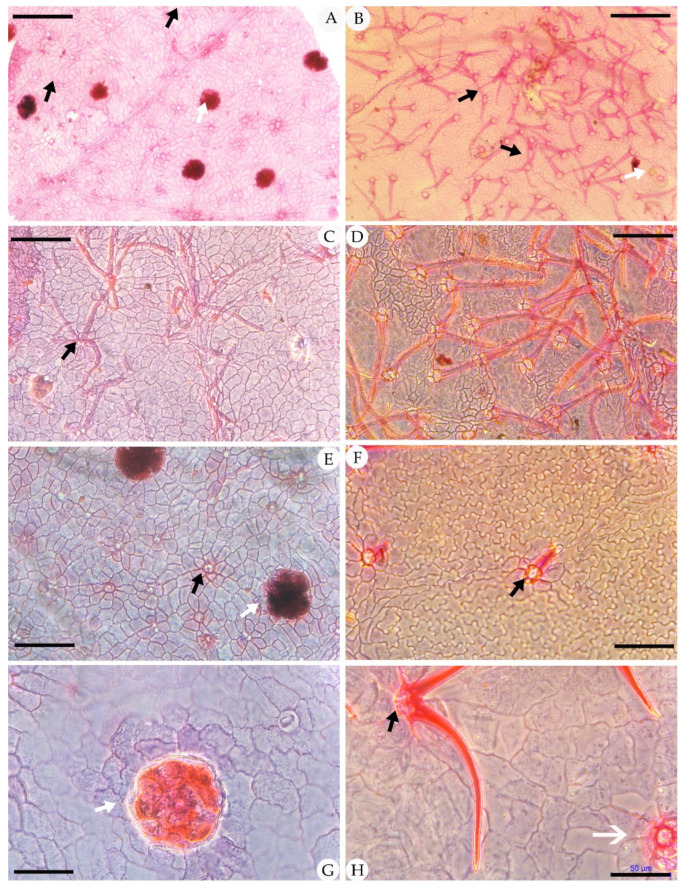
Adaxial cuticle of fossil *Comptonia hirsuta* and extant *C. peregrina.* (**A**) The simple, paired trichome bases and peltate glandular trichomes of *Comptonia hirsuta*, holotype, ELS-16-301. (**B**) The adaxial cuticle of *C. peregrina* from Massachusetts, USA, showing the dense trichomes with simple, paired, and four-celled fused trichome bases. (**C**) The adaxial cuticle of *Comptonia hirsuta*, showing a four-celled fused trichome base, ELS-16-196. (**D**) The dense trichomes of *C. peregrina* from Massachusetts, USA. (**E**) The simple trichome bases and peltate glandular trichomes of *Comptonia hirsuta*, holotype, ELS-16-301. (**F**) Undulate epidermic cells and simple trichomes of *C. peregrina* from New Hampshire, USA. (**G**) Slightly undulate epidermic cells and peltate glandular trichomes of *Comptonia hirsuta*, ELS-16-217. (**H**) The paired trichome bases and peltate glandular trichomes of *C. peregrina* from Virginia, USA. Black arrows show the trichome bases, and white arrows show the peltate glandular trichomes. Scale bar: (**A**,**B**) = 200 μm; (**C**–**F**) = 100 μm; (**G**,**H**) = 50 μm.

**Figure 4:**
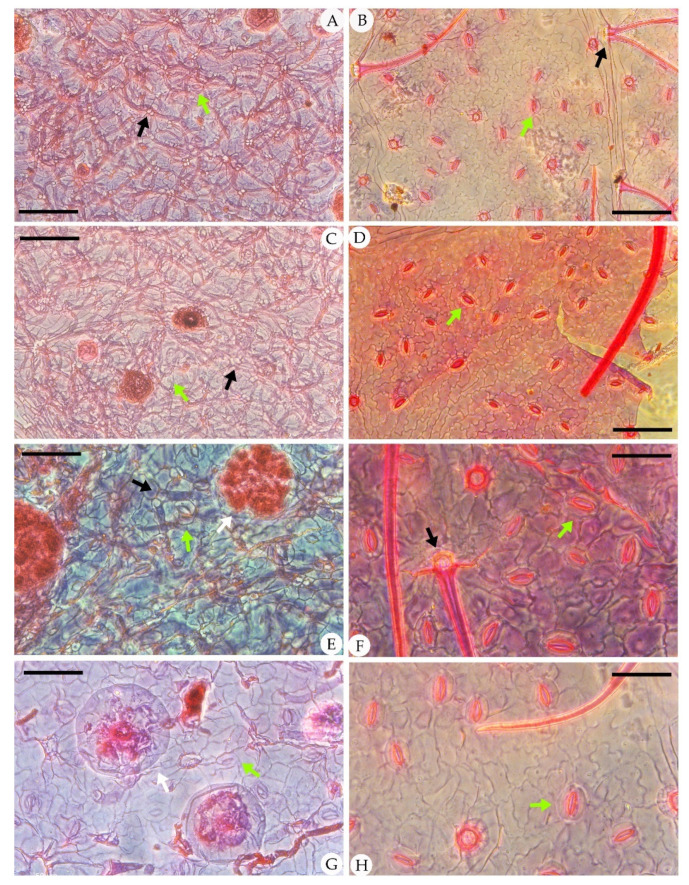
Abaxial cuticle of fossil *Comptonia hirsuta* and extant *C. peregrina.* (**A**) The dense trichomes with simple, paired, and four-celled fused trichome bases; peltate glandular trichomes; and anomocytic broadly elliptical stomata of *Comptonia hirsuta*, ELS-16-270. (**B**) The abaxial cuticle of *C. peregrina* from Virginia, USA, showing the simple, paired trichome bases. (**C**) The abaxial cuticle of *Comptonia hirsuta*, showing dense trichomes, peltate glandular trichomes, and anomocytic stomata, ELS-16-216. (**D**) The anomocytic stomata and undulate epidermic cells of *C. peregrina* from Maine, USA. (**E**) The paired trichome bases, peltate glandular trichomes, and anomocytic stomata of *Comptonia hirsuta*, holotype, ELS-16-301. (**F**) Slightly undulate epidermic cells, simple trichomes, and anomocytic stomata of *C. peregrina* from New Hampshire, USA. (**G**) Anomocytic stomata and peltate glandular trichomes of *Comptonia hirsuta*, ELS-16-146. (**H**) The anomocytic stomata and slightly undulate epidermic cells of *C. peregrina* from Virginia, USA. The black arrows show the trichome bases; green arrows point to the stomata; and the white arrows show the peltate glandular trichomes. Scale bar: (**A**–**D**) = 100 μm; (**E**–**H**) = 50 μm.

**Figure 5:**
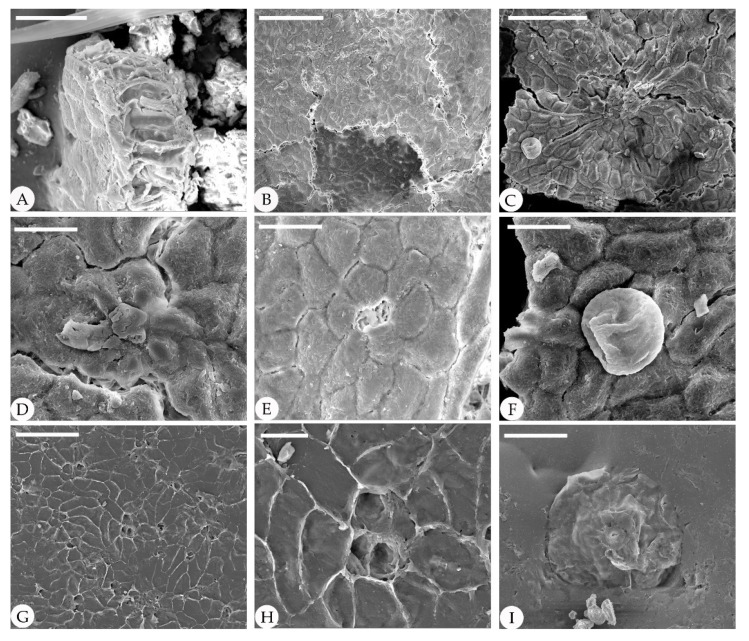
Cuticle of fossil *Comptonia hirsuta* under SEM, ELS-16-216. (**A**) Cross-section of the fossil leaf showing the palisade tissue cells. (**B**–**I**) are the structures on the adaxial cuticle. (**B**) Straight epidermic cells with trichome bases. (**C**) Straight epidermic cells with a peltate glandular trichome. (**D**) Trichome base. (**E**) Details of (**B**), showing a trichome base surrounded by six cells. (**F**) Details of (**C**), showing a peltate glandular trichome. (**G**) Straight epidermic cells with trichome bases. (**H**) Details of (**G**), showing a three-cell fused trichome base. (**I**) A peltate glandular trichome. Scale bar: (**A**) = 30 μm; (**B**,**C**) = 100 μm; (**D**–**F**) = 20 μm; (**G**) = 50 μm; (**H**) = 10 μm; (**I**) = 40 μm.

**Figure 6:**
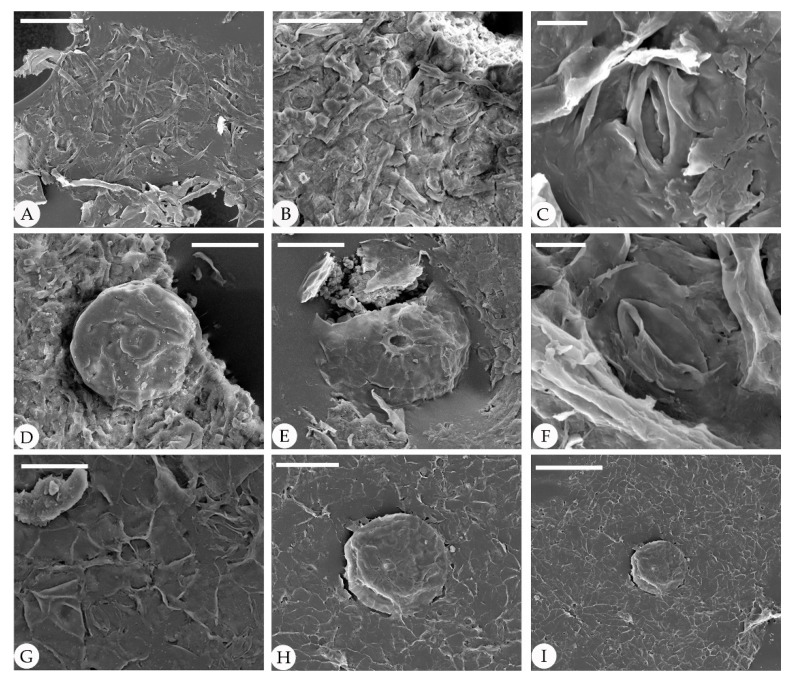
Abaxial cuticle of fossil *Comptonia hirsuta* under SEM, ELS-16-216. (**A**) Dense trichomes. (**B**) Anomocytic stomata. (**C**) Details of anomocytic stomata. (**D**) A peltate glandular trichome with a one-celled stalk. (**E**) A broken peltate glandular trichome. (**F**) Anomocytic stomata. (**G**) Paired trichome base and anomocytic stomata. (**H**) Details of (**I**), showing a peltate glandular trichome with a one-celled stalk. (**I**) A peltate glandular trichome and trichome bases of simple trichomes. Scale bar: (**A**,**C**,**F**,**H**) = 50 μm; (**B**,**D**,**E**) = 40 μm; (**G**) = 20 μm; (**I**) = 100 μm.

**Figure 7:**
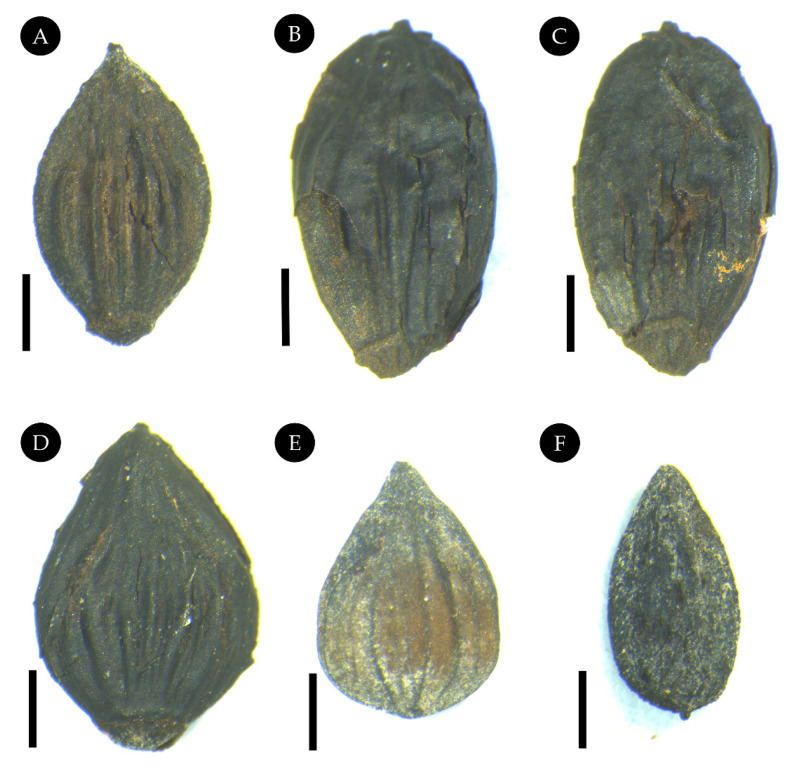
Fruits of *Comptonia hirsuta* from early Miocene of Zhuozi. (**A**) No. ELS-18-116. (**B**) No. ELS-18-205A. (**C**) No. ELS-18-205B. (**D**) No. ELS-18-154. (**E**) No. ELS-18-246. (**F**) No. ELS-18-232. Scale bar: (**A**–**F**) = 1 mm.

**Figure 8:**
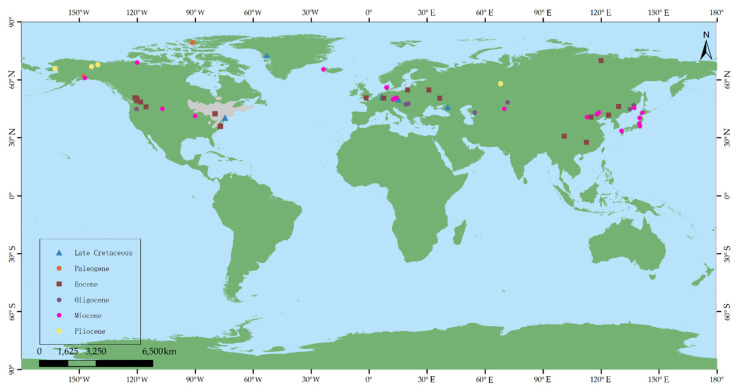
The fossil records and the present distribution range (represented in grey) of *Comptonia* in the world. Base maps were constructed with ArcGIS 10.2 based on data from the Resource and Environment Data Cloud Platform [53].

The global paleogeographic distribution of *Comptonia* has been researched in a general manner. However, the paleogeographic changes in China have not been studied in detail. In this research, we noticed that the genus *Comptonia* in particular has a conspicuous variation in its distribution in China (Figure 9).

In China, *Comptonia* fossils were found to occur in more widespread areas during the Eocene, which were in or near the humid zone of China (Figure 9A), such as Yilan, Heilongjiang [37]; Fushun, Liaoning [26,35,37]; Zhangjiakou, Hebei [26]; Litang, Sichuan [6]; and Xiangxiang, Hunan [26]. The warm climate of the Eocene, as well as the humid environment, provided good conditions for the survival of *Comptonia*. However, *Comptonia* fossils were not found in China during the Oligocene, possibly due to the poorer preservation of the Oligocene terrestrial strata relative to the Eocene strata and the Miocene strata, which were influenced by the uplift of some basins in the mainland of China [54]. Moreover, the global cooling event from the late Eocene to the Oligocene may also have influenced the distribution of *Comptonia.* In the Miocene, the distribution range of *Comptonia* is significantly reduced relative to that in the Eocene. During the Miocene, *Comptonia* remains were only found in the northern regions of China, such as Chifeng, Zhuozi of the Inner Mongolia Autonomous Region, and Weichang of Hebei (Figure 9C). This may be due to the apparent cooling event from the late Oligocene to the early Miocene [52] that had some impact on the survival of the genus. On the other hand, the geological age of the fossil site in Liangcheng, where *Comptonia* leaf fossils were reported by Zhang [55], is actually of the Miocene rather than the Pliocene, according to the investigation of stratigraphic correlations. In fact, after the Miocene, *Comptonia* fossils have hitherto not been found in China. However, the Pliocene strata is extensively exposed in China [54]. Thus, the preservation bias of *Comptonia* fossils should be excluded. An interesting question concerns the reason for the disappearance of *Comptonia* in China after the Miocene.

The vegetation types in Zhuozi, Chifeng, and Weichang during the Miocene period belong to mixed northern hardwood forests [54]. The climate of central Inner Mongolia during this period was warm and humid, similar to that of present-day eastern North America, and suitable for the growth of the *Comptonia* population. After the late Miocene, the global climate gradually cooled, leading to the global Great Ice Age of the Quaternary. Such drastic climatic change caused the vegetation type of the Inner Mongolia region to change from mixed northern hardwood forests to temperate grasslands. Moreover, central Inner Mongolia changed from being a humid area to a dry or semi-dry zone because of the aridification of the Chinese inland area. Tredici and Torrey [56] found that germination of *Comptonia* seeds is very difficult, meaning that *Comptonia* cannot reproduce annually by seed germination. In this case, root tillers became another important propagational pattern of *Comptonia* (i.e., new plantlets are formed by growing adventitious shoots on the roots that stick out of the ground), which would limit its dispersal scope. Moreover, the prolonged cooling and drying climates covering in the Inner Mongolia region after the late Miocene were not suitable for the survival of *Comptonia*. As a result, both climatic and progenitive factors may together have resulted in the disappearance of *Comptonia* in China.

## Data Availability

The data presented in this study are available on request from the corresponding author.
